# NMR Insights into the Structure-Function Relationships in the Binding of Melanocortin Analogues to the MC1R Receptor

**DOI:** 10.3390/molecules22071189

**Published:** 2017-07-15

**Authors:** Maurício Morais, Héctor Zamora-Carreras, Paula D. Raposinho, Maria Cristina Oliveira, David Pantoja-Uceda, João D. G. Correia, M. Angeles Jiménez

**Affiliations:** 1 Centro de Ciências e Tecnologias Nucleares, Instituto Superior Técnico, Universidade de Lisboa, Estrada Nacional 10 (km 139.7), 2695-066 Bobadela LRS, Portugal; m.morais@ucl.ac.uk (M.M.); paular@ctn.tecnico.ulisboa.pt (P.D.R.); cristinaoliveira@ctn.tecnico.ulisboa.pt (M.C.O.); jgalamba@ctn.tecnico.ulisboa.pt (J.D.G.C.); 2 Division of Imaging Sciences and Biomedical Engineering, King’s College London, 4th Floor Lambeth Wing, St Thomas’ Hospital, London SE1 7EH, UK; 3 Instituto de Química Física Rocasolano (IQFR), Consejo Superior de Investigaciones Científicas (CSIC), Serrano 119, 28006 Madrid, Spain; hzamora@iqfr.csic.es (H.Z.-C.); dpantoja@iqfr.csic.es (D.P.-U.)

**Keywords:** α-MSH analogue, cyclic peptide, melanocortin receptor, NMR, peptide structure

## Abstract

Linear and cyclic analogues of the α-melanocyte stimulating hormone (α-MSH) targeting the human melanocortin receptor 1 (MC1R) are of pharmacological interest for detecting and treating melanoma. The central sequence of α-MSH (His-Phe-Arg-Trp) has been identified as being essential for receptor binding. To deepen current knowledge on the molecular basis for α-MSH bioactivity, we aimed to understand the effect of cycle size on receptor binding. To that end, we synthesised two macrocyclic isomeric α-MSH analogues, c[NH-NO_2_-C_6_H_3_-CO-His-DPhe-Arg-Trp-Lys]-Lys-NH_2_ (**CycN-K6**) and c[NH-NO_2_-C_6_H_3_-CO-His-DPhe-Arg-Trp-Lys-Lys]-NH_2_ (**CycN-K7**). Their affinities to MC1R receptor were determined by competitive binding assays, and their structures were analysed by ^1^H and ^13^C NMR. These results were compared to those of the previously reported analogue c[S-NO_2_-C_6_H_3_-CO-His-DPhe-Arg-Trp-Cys]-Lys-NH_2_ (**CycS-C6**). The MC1R binding affinity of the 22-membered macrocyclic peptide **CycN-K6** (IC_50_ = 155 ± 16 nM) is higher than that found for the 25-membered macrocyclic analogue **CycN-K7** (IC_50_ = 495 ± 101 nM), which, in turn, is higher than that observed for the 19-membered cyclic analogue **CycS-C6** (IC_50_ = 1770 ± 480 nM). NMR structural study indicated that macrocycle size leads to changes in the relative dispositions of the side chains, particularly in the packing of the Arg side chain relative to the aromatic rings. In contrast to the other analogues, the 22-membered cycle’s side chains are favorably positioned for receptor interaction.

## 1. Introduction

The melanocortin receptor 1 (MC1R) belongs to a five-member subfamily of G protein-coupled receptors (GPCR), the melanocortin receptors (MCRs) family that also includes MC2-, MC3-, MC4- and MC5-receptors [1,2,3,4,5]. The human gene coding the MC1R was first cloned in 1992 [1,6]. Since then, genetic analysis, phenotypic association and structure-function studies opened new perspectives for the assignment of its function as a key regulator of skin biological events. It is involved in the regulation of skin pigmentation, animal coat coloration, and melanocyte function [5,7]. Polymorphisms in the receptor gene have been attributed to the red hair phenotype, melanoma and non-melanoma skin cancer [7,8,9]. MC1R is expressed in melanocytes, melanoma cells, macrophages, and brain, as well as in leukocytes, where it may mediate an anti-inflammatory action [5,10]. MC2R is expressed in the adrenal cortex, where it mediates the effects of the adrenocorticotropic hormone (ACTH) on steroid secretion. MC3R, found in many areas of the central nervous system and peripheral tissues, modulates energy homeostasis. MC4R is expressed predominantly in the central nervous system and regulates both food intake and sexual function, while MC5R is expressed in numerous human peripheral tissues and is mainly involved in exocrine function, particularly sebaceous gland secretion [5,11].

The MC1R shares the same endogenous ligands with other MCRs, and, therefore should have similar orthosteric binding (i.e., the receptor area where the endogenous ligand binds) [5]. The orthosteric binding site is located in the transmembrane (TM) helices of MC1R, and several negatively-charged, aromatic and aliphatic residues have been identified as crucial for ligand binding. MC1R binds to several natural ligands, which are derived from the same precursor protein (pro-opiomelanocortin—POMC), with the following order of potency: α-melanocyte-stimulating hormone (α-MSH) > ACTH > β-MSH > γ-MSH [7,12].

Among the reported signaling pathways, it is noteworthy that MC1R mediates the physiological actions of the endogenous ligand by a Gs-protein-dependent activation of the cyclic adenosine monophosphate (cAMP) signaling pathway. The increase of cAMP levels results in the activation of tyrosinase in melanocytes and leads to an increase of melanin production [11]. This mechanism of production and accumulation of intracellular cAMP has been used to determine the agonist/antagonist properties of MC1R ligands [11].

Structure–bioactivity studies have shown that all endogenous ligands contain a conserved His-Phe-Arg-Trp sequence that has been attributed to melanocortin receptor selectivity and stimulation [5,7,12,13,14]. This high degree of pharmacophore homology makes it difficult to design highly selective ligands for one receptor subtype. Nevertheless, MC1R is known to be expressed at the surface of human melanocytes, and overexpressed at the surface of murine and human melanoma cells, while MC2R-MC5R has higher expression levels in the brain and the nervous system. Thus, MC2R-MC5R off-target interactions are unlikely because presumably most ligands will not be able to cross the blood-brain barrier [15,16,17]. Taken together, these findings supported the development of strategies to specifically target MC1R without significant side effects.

Among these strategies, a great deal of research has focused on the design of α-MSH peptide analogues because the receptor participates in several signaling pathways and since non-peptide or small ligands are not available to mimic the physiological action of the linear tridecapeptide α-MSH [11].

**Figure 1:**
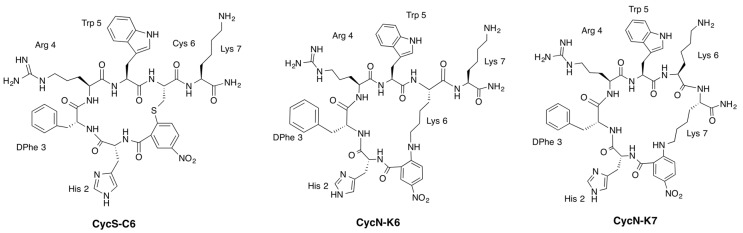
Structural formulas of the cyclic α-melanocyte stimulating hormone (α-MSH) analogues **CycS-C6**, **CycN-K6**, and **CycN-K7**.

Aimed at enhancing the biological stability, potency and specificity of the endogenous ligand, a library of α-MSH analogues has been designed for imaging and therapeutic applications [14,18]. Among them, the 19-membered alkylthioaryl-bridged macrocyclic α-MSH analogue c[S–NO_2_–C_6_H_3_–CO–His–DPhe–Arg–Trp–Cys]–NH_2_ (**PG10N**) was reported as a potent full agonist for all human MCRs [19]. Recently, we found that the addition of a Lys at the C-terminus of the parent peptide **PG10N** led to impaired binding of analogue **CycS-C6** (Figure 1 and Table 1). However, replacement of the alkylthioaryl-bridge (**CycS-C6**) by an alkylaminoaryl-bridge (**CycN-K6**) led to a 22-membered macrocyclic peptide with increased affinity towards MC1R (Table 1 and Figure 1). 

These observations, which suggest that the size of the macrocycle might play a role for receptor binding, prompted us to experimentally analyse the structural factors that may affect the MC1R-interaction properties of these macrocyclic α-MSH analogue peptides. Since we have already reported the NMR structure of the alkylthioaryl-bridged analogue **CycS-C6** [20], we proceeded to perform the NMR structural study of the alkylaminoaryl-bridged macrocyclic peptide **CycN-K6** in aqueous solution. Furthermore, we examined the affinity for receptor MC1R and extended the NMR study to the 25-membered alkylaminoaryl-bridged macrocyclic peptide **CycN-K7** (Figure 1). This peptide, initially obtained as a sub-product in the synthesis of **CycN-K6**, is interesting because of its larger macrocycle size and since it is an isomer of **CycN-K6**.

## 2. Results

### 2.1. Affinity of Peptides **CycN-K6** and **CycN-K7** to MCR1

The binding affinities of the peptides **CycN-K6** and **CycN-K7** to MC1R were tested in a competitive binding assay using [^125^I]-[Nle^4^,DPhe^7^]-α-MSH ([^125^I]-NDP-α-MSH) as radioligand and murine melanoma B16F1 cells (Figure S1, Supplementary Materials), as previously described for other α-MSH analogues [20]. Table 1 lists the IC_50_ values obtained for those two peptides, and for the 19-membered macrocyclic peptides **PG10N** and **CycS-C6** [19,20]. Strikingly, the 25-membered peptide **CycN-K7** turned out to have less affinity for the receptor than the 22-membered peptide **CycN-K6**, which, however, exhibits more affinity than the 19-membered peptide **CycS-C6**. This indicates that the relationship between cycle size and receptor affinity is not straightforward. Therefore, a detailed structural comparison of these three peptides is required to understand the molecular basis for their binding to receptor MC1R.

### 2.2. NMR Characterization of **CycN-K6** and **CycN-K7** and Comparison to **CycS-C6**

**Figure 2:**
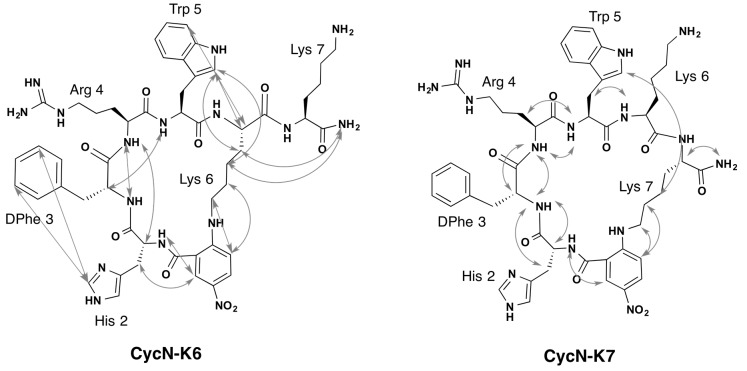
Relevant NOEs observed for peptides **CycN-K6** and **CycN-K7** in aqueous solution. The double arrows connect the protons for which NOE cross-peaks are observed in the ^1^H,^1^H-ROESY spectra.

Peptides **CycN-K6** and **CycN-K7** are structural isomers, which differ in the Lys side chain involved in cyclization (Figure 1). Once their chemical shifts (δ) were assigned (see Section 4 and Tables S1 and S2, Supplementary Materials), the identity of the Lys residue involved in cyclization could be confirmed unambiguously by ^1^H and ^13^C δ values of the Lys C_εε’_H_2_ groups (Tables S1 and S2), and by the Nuclear Overhauser effects (NOEs) between the *p*-NO_2_-benzoic acid ring and the Lys side chain (Figure 2 and Table S3, Supplementary Materials).

**Figure 3:**
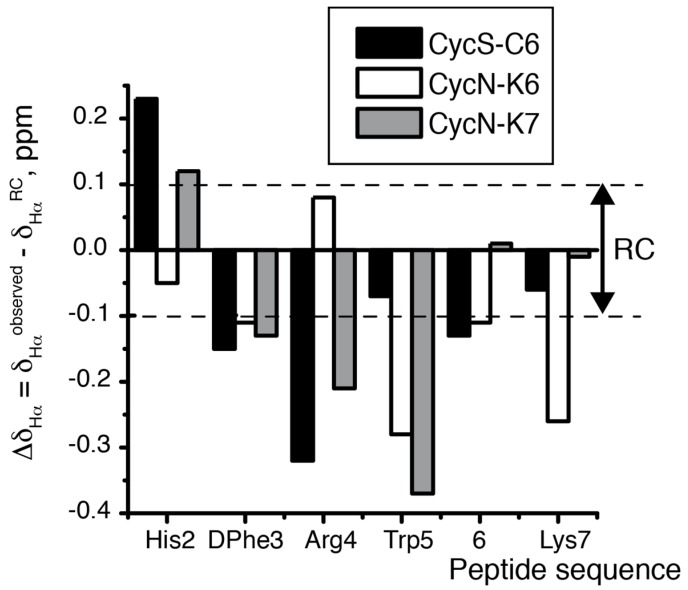
Δδ_Hα_ (Δδ_Hα_ = δ_Hα_^observed^ − δ_Hα_^RC^, ppm) plot as a function of sequence for peptides **CycS-C6** (black bars), **CycN-K6** (white bars), and **CycN-K7** (grey bars) in aqueous solution at pH 2.5 and 25 °C. Residue 6 is Cys in **CycS-C6** and Lys in **CycN-K6** and **CycN-K7**. Dashed lines indicate the random coil (RC) range. Random coil values were taken from [21].

As previously found in peptide **CycS-C6** [20], these two macrocyclic peptides show many δ values that differ strongly from the random coil δ values. For instance, the chemical shift deviations (Δδ = δ_observed_ − δ_random coil_, ppm) obtained for ^1^H_α_, ^13^C_α_ and ^13^C_β_ atoms lie clearly outside the random coil range (Figure 3 and Figure S2, Supplementary Materials), which shows that peptides **CycN-K6** and **CycN-K7** form ordered conformations in aqueous solution. Moreover, the sign of the observed deviations, which are negative in Δδ_Hα_ and Δδ_Cβ_, and positive in Δδ_Cα_ for most residues (Figure 3 and Figure S2), is indicative of turn-like conformations. Concerning side chain protons, the up-field shifts exhibited by Arg (Table 2) indicate that these side chain protons experience anisotropic effects from the ring currents of one or more aromatic residues, as was also observed in peptide **CycS-C6** [20]. Despite these common general characteristics, the detailed examination of NMR parameters highlights the existence of important differences between peptides **CycN-K6** and **CycN-K7**, and also among these peptides and the previously reported **CycS-C6** [20]. The differences are observed both at the cycle backbone and at certain side chains. The most remarkable differences at cycle backbone are observed for residues His and Arg, for which peptide **CycN-K6** exhibits Δδ_Hα_ values of opposite sign relative to those observed in peptides **CycS-C6** and **CycN-K7** (Figure 3 and Table 2). In addition, ^3^J_αN_ coupling constants, which have a well-established relationship with φ angles, displayed differences among the three peptides. DPhe residue shows a small ^3^J_αN_ value (<5 Hz) in peptide **CycS-C6** [20], which is typical of the i + 1 residue in β-turns, but values about 6–7 Hz in peptides **CycN-K6** and **CycN-K7** (Table 2). In contrast, the ^3^J_αN_ value is small for the His residue in peptide **CycN-K6**, and displays a large value, which is characteristic of extended chains, in peptide **CycS-C6** (Table 2). This suggests that the residues forming a β-turn structure differ between peptide **CycS-C6** and **CycN-K6**. No residue shows a small value in the case of peptide **CycN-K7** (Table S4), probably because its larger macrocyclic size imposes less restrictions on mobility. It seems obvious that the differences in macrocycle conformations should translate into changes in the relative dispositions of the side chains. Thus, the magnitudes of the Arg up-field shifted ^1^H δ differ significantly among the three peptides, and follow the decreasing order **CycS-C6** > **CycN-K7** > **CycN-K6** (Table 2). These differences must arise from changes in the packing of the Arg side chain relative to the aromatic rings, as confirmed in the calculated structures (see below). All these conformational variations account for the differences in affinity to receptor MC1R (Table 1).

### 2.3. NMR Solution Structures of Analogues **CycN-K6** and **CycN-K7**

**Figure 4:**
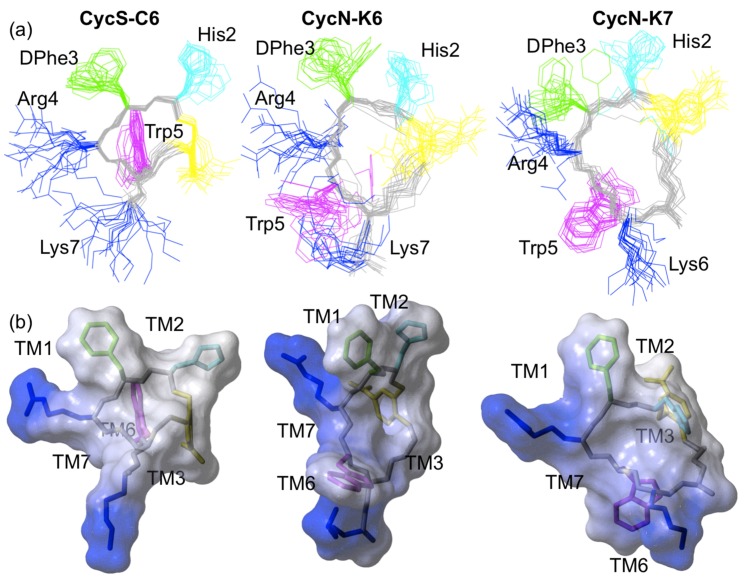
3D NMR structures of peptides **CycS-C6** [20], **CycN-K6** and **CycN-K7**. (**a**) Ensemble of the 20 lowest target function calculated structures. Side chains are colored in cyan for His, green for DPhe, magenta for Trp, and blue for Arg and Lys. Backbone atoms are displayed in grey and the thioaryl-bridge in yellow. (**b**) Electrostatic surface of a representative conformer of each peptide overlaid onto the corresponding structure. Blue color indicates positively-charged areas. The MC1R transmembrane helices (TM1, TM2, TM3, TM6 and TM7), which are close to the essential peptide side chains (His, DPhe, Arg and Trp) in the reported MC1R/peptide model [24], are indicated. In the represented views, TM6 and TM3 lie behind **CysS-C6** and **CycN-K7**, respectively.

According to the qualitative analysis of the NMR data, peptides **CycS-C6** [20], **CycN-K6** and **CycN-K7** present some conformational differences. The size of the macrocycle seems to affect the cycle conformation, which, in turn, induces changes in the relative positions of aromatic and positively-charged side chains. To achieve a detailed comparison of the three-dimensional arrangement of the side chains in the three peptides, we proceeded to calculate the structures of peptides **CycN-K6** and **CycN-K7** from the experimental NMR parameters (see Materials and methods). The solution structure of **CycS-C6** had been previously determined following the same calculation protocol [13]. The structural ensembles of the three peptides are represented in Figure 4. Peptide **CycS-C6**, which has the smallest cycle size (19 atoms), has the lowest RMSD value (0.4 Å). Thus, as expected, the smaller macrocycle restricts the most the available conformations. However, **CycN-K7**, which has the biggest cycle size (25 atoms), has a slightly lower RMSD (0.7 Å) than peptide **CycN-K6** (0.8 Å), whose cycle size (22 atoms) is intermediate between **CycS-C6** and **CycN-K7**. Although the difference in RMSD value between **CycN-K6** and **CycN-K7** is very small, it is interesting to note that increasing or decreasing the cycle size in the antimicrobial peptide Gramicidin S results in a well-defined antiparallel β-sheet or a less-defined distorted structure depending on the number of cycle residues [23]. Apart from this, the main differences among the structures lie in the relative position of the side chains (Figure 4), in particular the Trp side chain shows quite a different orientation in the three peptides (Figure S3, Supplementary Materials). These differences should account for the distinct affinities to MC1R receptor exhibited by the three peptides.

## 3. Discussion

Based on sequence homology, the structure of the MC1R receptor consists of seven transmembrane helices, TM1 to TM7 [12,25], which are very probably arranged in the helical bundle characteristic of the GPCR superfamily. In the proposed model for the complex between the human MC1R receptor and a rigid cyclic melanocyte-stimulating hormone core peptide c[His–DPhe–Arg–Trp–Gly], the ligand lies into an inner channel of the helical bundle, and close to the extracellular loops. In that model [24], the guanidinium group of the Arg residue interacts with a cluster of negatively-charged residues and polar residues at TM1 and TM7 helices; the DPhe aromatic ring lies in a hydrophobic cavity between TM1 and TM2 helices; the Trp indole moiety interacts with a hydrophobic cluster formed by residues from TM3, TM6, and TM7 helices, and it might be hydrogen-bonded to an Asp at TM3 helix; and the His imidazole group points towards the extracellular loops and contacts with residues at TM2 helix. Helices TM4 and TM5 do not participate in the binding [24,26].

Considering this complex network of interactions, the structural differences among the three peptides (Figure 4 and Figure S3, Supplementary Materials) can explain their distinct affinity to MC1R (Table 1). As we previously reported [20], the extra-cyclic Lys present in **CycS-C6** strongly decreases affinity to MC1R (Table 1). Likely, this additional long and positively-charged Lys side chain impedes suitable accommodation of the side chains of the cycle residues at the MC1R binding pocket. The extra-cyclic Lys might interfere either by steric hindrance or by competing with the Arg binding site. Strikingly, the MC1R affinity of **CycN-K6**, which also has an extra-cyclic Lys, but a larger cycle, is about ten-fold higher than in **CycS-C6** (Table 1). Indeed, this result prompted us to characterize the structure of peptide **CycN-K6** (see Introduction). As we hypothesized [20], the differences in the size of the cycle give rise to different relative arrangements of all the side chains. These differences can be appreciated for the His/DPhe, DPhe/Arg, Arg/Trp, and DPhe/Trp pairs in Figure 4. For example, the aromatic rings of DPhe3 and Trp5 extend outwards from the same macrocycle face in **CycS-C6** (61 ± 24°) but from different sides in **CycN-K6** (99 ± 38°). Considering that DPhe and Trp aromatic rings bind to different sub-sites in the MC1R receptor [24], it seems likely that these rings’ position on different cycle faces in **CycN-K6** will predispose them to interact favorably with MC1R. In contrast, the alignment of these aromatic rings on the same cycle face in **CycS-C6** predisposes them unfavorably for binding, and it can be expected that part of the **CycS-C6**/MC1R binding energy will have to be “spent” to change their orientation. These expectations are supported by the higher MC1R affinity of **CycN-K6** compared to **CycS-C6** (Table 1). In the case of peptide **CycN-K7**, which shows an intermediate affinity value between the other two peptides, the orientation of the DPhe and Trp rings (69 ± 20°) is more similar to **CycS-C6** than **CycN-K6** (see also Figure S3, Supplementary Materials).

Analogously, the other residue pairs are probably better predisposed in **CycN-K6** to interact with the MC1R receptor. For instance, it can be seen in Figure 4 that the Arg/DPhe and DPhe/His pairs display quite different arrangements in the three peptides. Since the DPhe aromatic ring interacts with residues at the TM1 and TM2 helices, Arg with residues at TM1, and His with residues at TM2, positioning these three side chains in a conformation that resembles their orientation when bound to the MC1R receptor should facilitate complex formation. Based on this line of reasoning, we can infer that the melanocortin core residues (His, DPhe, Arg, and Trp) in **CycN-K6** are favorably arranged to bind the MC1R receptor.

Evidently, the conformation of the free and MC1R-bound peptides could differ. Then, considering peptides whose side chains are not favorably predisposed to bind the receptor, we can expect that flexible peptides will bind better than rigid ones. This is because less binding energy will be “spent” forcing a flexible peptide to adopt the proper orientation as compared to a rigid one. Indeed, some cases in which the most flexible variant of a peptide is more active than those more structurally restricted have been reported [27,28]. Although RMSD value is not a dynamic parameter, in general, low RMSD values correspond to well-defined, and usually mostly rigid structures, whereas high RMSD values are found for less-defined, and more-flexible conformers. Accordingly, the structure of the 19-membered macrocycle **CycS-C6** is better defined, and therefore is likely to be more rigid, than those of the 22- and 25-macrocycles **CycN-K6**, and **CycN-K7**, respectively. Hence, if receptor binding requires structural re-arrangements, these two peptides will adapt more easily than **CysS-C6**. This explains the lower affinity for MC1R by **CysS-C6** in comparison with **CycN-K7**, even though the side chains of these two peptides dispose differently from **CycN-K6**, and more similarly between them.

Coming back to the Lys residue, which is present in the three peptides, and strongly decreases affinity to MC1R (see above **CysS-C6** vs. **PG10N**; Table 1); the distribution of the positively-charged residues (Arg4 and Lys6/7; Figure 4) differs among the three peptides. Thus, the fact that the detrimental effect of Lys is not so drastic in **CycN-K6**, and **CycN-K7** could be explained by its disfavoring effect being offset by other favorable factors, such as a suitable pre-arrangement of the side chains in **CycN-K6**; and by the higher flexibility of these larger macrocycles, which makes feasible for the Lys side chain to be placed so that it does not disturb the favorable key interactions of the conserved melanocortin core residues. The conformational space available for Lys6 in **CycN-K7**, which is within the cycle, must be more restricted than for Lys7 in **CycN-K6**, which is outside the cycle (Figure 1 and Figure 4), and likely contributes to the lower MC1R affinity of **CycN-K7** relative to **CycN-K6** (Table 1).

Taken together, our results have shown that appropriate side chain orientations, as proposed by Cho and coworkers [29], and conformational flexibility play an important role in the binding affinity to melanocortin receptors. Upon the increase of the cycle size, the side chain orientations and the cycle flexibility are changed, and as a consequence binding affinity is affected.

## 4. Materials and Methods 

### 4.1. Peptide Synthesis

Peptides c[NH–NO_2_–C_6_H_3_–CO–His–DPhe–Arg–Trp–Lys]–Lys–NH_2_ (**CycN-K6**) and c[NH–NO_2_–C_6_H_3_–CO–His–DPhe–Arg–Trp–Lys–Lys]–NH_2_ (**CycN-K7**) were prepared following modified protocols from the literature [19]. Details for peptide **CycN-K6** were previously reported [20]. In the case of peptide **CycN-K7**, the linear peptide NH_2_–His(Trt)–DPhe–Arg(Pbf)–Trp(Boc)–Lys(ivDde)–Lys(Mtt)–NH_2_ was assembled to the MBHA Rink Amide resin (Novabiochem, Lisbon, Portugal) by Fmoc-based solid-phase peptide synthesis in a CEM 12-channel automated peptide synthesizer (CEM Corporation, Matthews, NC, USA) using 1-hydroxibenzotriazole hydrate (HOBT)/*O*-(benzotriazole-1-yl)-*N*,*N*,*N*′,*N*′-tetramethyluronium hexafluorophosphate (HBTU) as coupling agents, and capped with fluoronitrobenzoic acid as previously described [19]. Prior to the cyclization step, the NH-Mtt protecting group of Lys was removed with dilute TFA (1% in CH_2_Cl_2_) without cleavage of the peptide from the resin. As described for peptides **CycS-C6** and **CycN-K6** [20], cyclization was performed by treating the supported peptide with 5 equiv. of K_2_CO_3_ in DMF at 25 °C with gentle shaking for 36 h. Next, the peptide resin was washed with DMF (2×), H_2_O (3×), DMF (3×), H_2_O (2×), CH_3_OH (3×), and CH_2_Cl_2_ (3×) and then vacuum-dried. Cleavage of the peptide from the resin was accomplished by treatment with a mixture of 95 % TFA, 2.5 % TIS, and 2.5 % H_2_O (5 mL) for 2 h. The cleavage solution, which contains the crude peptide, was separated from the resin by filtration and concentrated by partial evaporation of the solvent using a stream of N_2_ gas The crude peptide **CycN-K7** was precipitated and washed with diethyl ether, vacuum-dried, and dissolved in H_2_O prior to lyophilisation. Finally, semi-preparative purification and evaporation of solvents from the corresponding fractions yielded peptide **CycN-K7** as a green solid.

Peptide **CycN-K6**, c[NH–NO_2_–C_6_H_3_–CO–His–DPhe–Arg–Trp–Lys]–Lys–NH_2_ (MW C_51_H_66_N_16_O_9_ 1047.1): calculated *m/z* for [M + 2H]^2+^ 524.5, found 524.5, t_R_ = 9.3 min.

Peptide **CycN-K7**, c[NH–NO_2_–C_6_H_3_–CO–His–DPhe–Arg–Trp–Lys–Lys]–NH_2_ (MW C_51_H_66_N_16_O_9_ 1047.1): calculated *m/z* for [M + 2H]^2+^ 524.5, found 524.5, t_R_ = 9.5 min.

### 4.2. Competitive Binding Assays

NDP-α-MSH was radioiodinated as described [20]. To determine the inhibitory concentration of 50 % (IC_50_) values for the α-MSH analogues, competitive binding assays with [^125^I]NDP-α-MSH in B16F1 melanoma cells were carried out in triplicate following the previously described procedure [20]. IC_50_ values were calculated from the plots of the percentage of [^125^I]NDP-α-MSH bound to the cells vs. the concentrations of displacing peptides by using GraphPad Prism software (GraphPad Software Inc., La Jolla, CA, USA).

### 4.3. NMR Sample Preparation

Samples for acquisition of NMR spectra at approximately 1 mM peptide concentration were prepared by solving the lyophilized peptides (~0.5 mg) in 0.5 ml H_2_O/D_2_O (9:1 *v/v*). Sodium-2,2-dimethyl-2-silapentane-5-sulfonate (DSS) was added as an internal reference. A glass microelectrode was used to measure pH, which was adjusted to 2.5 by adding minimal amounts of NaOD or DCl. The pH readings were not corrected for isotope effects.

### 4.4. Acquisition of NMR Spectra

A Bruker (Bruker Española S.A, Madrid, Spain) AV-600 spectrometer operating at a proton frequency of 600.13 MHz and equipped with a cryoprobe was used to acquire NMR spectra. The temperature of the NMR probe was calibrated using a methanol sample. 1D and 2D spectra, i.e., ^1^H,^1^H-COSY (phase-sensitive correlated spectroscopy), ^1^H,^1^H-TOCSY (total correlated spectroscopy), and ^1^H,^1^H-ROESY (rotating frame nuclear Overhauser spectroscopy) were recorded and processed as previously reported [20,30]. ^1^H–^13^C and ^1^H–^15^N heteronuclear single quantum coherence spectra (HSQC) were acquired at natural abundance of the heteronuclei. ^13^C and ^15^N δ-values were indirectly referenced by multiplying the spectrometer frequency that corresponds to 0 ppm in the ^1^H spectrum, assigned to internal DSS reference, by 0.25144953 and 0.101329118, respectively [31].

### 4.5. NMR Spectral Assignment

^1^H resonances were assigned by analysis of 2D ^1^H,^1^H-COSY, ^1^H,^1^H-TOCSY, and ^1^H,^1^H-ROESY spectra using the program Sparky [32] and following standard sequential assignment methods [33,34]. Next, the ^13^C and ^15^N resonances were straightforwardly assigned from the cross-correlations observed in the corresponding HSQC spectra between the proton and the bound carbon or nitrogen, respectively. Supplementary Tables S1 and S2 list all chemical shifts for peptides **CycN-K6** and **CycN-K7**. The ^3^J_αN_ coupling constants were measured at the doublet signals observed for the HN amide protons in the 1D ^1^H-NMR spectra acquired in aqueous solution at pH 2.5 and 25 °C. They are listed at Supplementary Table S3.

### 4.6. Structure Calculation

The three dimensional structures were calculated from distance restraints derived from the cross-peaks present in ^1^H,^1^H-ROESY spectra of the peptides **CycN-K6** and **CycN-K7** (Figure S4) and using torsion angle dynamics as implemented in the CYANA program (P. Guntert’s group, Institute of Biophysical Chemistry, Goethe-University, Frankfurt am Main, Germany) [35,36]. The peak lists for ^1^H,^1^H-ROESY spectra were generated by interactive peak picking, and the peak volumes obtained by the automatic integration function of Sparky [32]. The CYANA protocol consisted of seven iterative cycles of automatic assignment of distance restraints and structure calculation, followed by a final standard structure calculation. In addition, a negative value for the torsion angle phi for all amino acids, except for DPhe, plus the distance restraints needed for cyclization between Lys6 or Lys7 and the *p*-NO_2_-benzoic acid linker were used during the NOE assignment/structure calculation cycles. In each cycle, the structure calculation started from 100 randomized conformers and the standard CYANA-simulated annealing schedule was used with 10,000 torsion angle dynamics steps per conformer. The final structures, which are the ensemble of the 20 structures with the lowest target functions, were visualised and analysed using the MOLMOL program [37].

## Figures and Tables

**Table 1:** Sequences, cycle size and MC1R binding affinities (IC_50_) of cyclic α-MSH analogues.

Peptide	Sequence	Cycle Size	IC_50_ (nM)
**PG10N** ^1^	c[S–NO_2_–C_6_H_3_–CO–His–DPhe–Arg–Trp–Cys]–NH_2_	19	3.7 ± 0.5
**CycS-C6** ^1^	c[S–NO_2_–C_6_H_3_–CO–His–DPhe–Arg–Trp–Cys]–Lys–NH_2_	19	1770 ± 480
**CycN-K6**	c[NH–NO_2_–C_6_H_3_–CO–His–DPhe–Arg–Trp–Lys]–Lys–NH_2_	22	155 ± 16
**CycN-K7**	c[NH–NO_2_–C_6_H_3_–CO–His–DPhe–Arg–Trp–Lys–Lys]–NH_2_	25	495 ± 101

^1^ Data for **PG10N** and for **CycS-C6** were taken from [19,20], respectively.

**Table 2:** Distinctive NMR parameters in peptides **CycS-C6**, **CycN-K7**, and **CycN-K6** ^1^.

	CycS-C6 ^2^	CycN-K6	CycN-K7	Random Coil ^3^
Cycle size (number atoms)	19	22	25	
^3^J_αN_ His (Hz) ^4^	7.9	4.2	6.0	5.5–7.4 ^5^
^3^J_αN_ DPhe (Hz) ^4^	4.8	6.6	6.2	5.5–7.4 ^5^
δ H_α_ His (ppm)	4.96	4.68	4.85	4.73
δ H_α_ Arg (ppm)	3.99	4.42	4.13	4.34
δ H_ββ’_ Arg (ppm)	1.12, 1.38	1.48, 1.69	1.41, 1.60	1.76, 1.86
δ H_γγ’_ Arg (ppm)	0.59, 0.79	1.02, 1.02	0.94, 1.08	1.63, 1.63
δ H_δδ’_ Arg (ppm)	2.78, 2.78	2.48, 2.82	2.71, 2.85	3.20, 3.20

^1^ Experimental conditions: H_2_O/D_2_O 9:1 *v/v* at pH 2.5 and 25 °C. ^2^ Data taken from [20]. ^3^ Values taken from [21]. ^4^ Experimental errors in ^3^J_αN_ are ± 0.4 Hz. ^5^ Range reported for non-structured model pentapeptides [22].

## Supplementary Materials

The following are available online, Figure S1: Competitive binding curves for **CycN-K6** and **CycN-K7**, Figure S2: Bar plots showing the Δδ_Cα_ and Δδ_Cβ_ values as a function of sequence for **CycS-C6**, **CycN-K6**, and **CycN-K7**, Figure S3: Overlay of representative structures for **CycS-C6**, **CycN-K6**, and **CycN-K7** highlighting the relative dispositions of DPhe, Arg and Trp side chains, Figure S4: ^1^H,^1^H-ROESY spectral region of **CycN-K6** and **CycN-K7**, Table S1: ^1^H, ^13^C and ^15^N chemical shifts for **CycN-K6**, Table S2: ^1^H, ^13^C and ^15^N chemical shifts for **CycN-K7**, Table S3. NOEs between *p*-NO_2_-benzoic acid unit and its adjacent residues His and Lys in **CycN-K6** and **CycN-K7**, Table S4. ^3^J_αN_ coupling constants measured for **CycS-C6**, **CycN-K6** and **CycN-K7**, Table S5. Structural statistics for the solution NMR structures of **CycN-K6** and **CycN-K7**.
