# Supplementary material for: NMR Insights into the Structure-Function Relationships in the Binding of Melanocortin Analogues to the MC1R Receptor
Source: Molecules. 2017 Jul 15;22(7):1189. doi: 10.3390/molecules22071189 (PMC6152105; doi:10.3390/molecules22071189)
Supplement: Supplementary file 1 [file molecules-22-01189-s001.pdf]

Supplementary Materials
for
NMR insights into the structure-function relationships in the binding of melanocortin analogues to the MC1R receptor

Maurício Morais [1,2], Héctor Zamora-Carreras [3], Paula D. Raposinho [1], Maria Cristina Oliveira [1], David Pantoja-Uceda [3], João D. G. Correia [1], and M. Angeles Jiménez [3,*]

[1] Centro de Ciências e Tecnologias Nucleares, Instituto Superior Técnico, Universidade de Lisboa, Estrada Nacional 10 (km 139,7), 2695-066 Bobadela LRS, Portugal
[2] Current address: Division of Imaging Sciences and Biomedical Engineering, King's College London, 4th Floor Lambeth Wing, St Thomas' Hospital, London SE1 7EH, UK
[3] Instituto de Química Física Rocasolano (IQFR), Consejo Superior de Investigaciones Científicas (CSIC), Serrano 119, 28006 Madrid, Spain
* Correspondence: majimenez@iqfr.csic.es

List of contents:

Figure S1: Competitive binding curves for **CycN-K6** and **CycN-K7**

Figure S2: Bar plots showing the $\Delta\delta_{C\alpha}$ and $\Delta\delta_{C\beta}$ values as a function of sequence for **CycS-C6**, **CycN-K6**, and **CycN-K7**

Figure S3: Overlay of representative structures for **CycS-C6**, **CycN-K6**, and **CycN-K7** highlighting the relative dispositions of DPhe, Arg and Trp side chains

Figure S4: ^{1}H,^{1}H-ROESY spectral region of **CycN-K6** and **CycN-K7**

Table S1: ^{1}H, ^{13}C and ^{15}N chemical shifts for **CycN-K6**

Table S2: ^{1}H, ^{13}C and ^{15}N chemical shifts for **CycN-K7**

Table S3: NOEs between pNO_2-benzoic acid unit and its adjacent residues His and Lys in **CycN-K6** and **CycN-K7**

Table S4: $^3J_{\alpha N}$ coupling constants measured for **CycS-C6**, **CycN-K6** and **CycN-K7**

Table S5: Structural statistics for the solution NMR structures of **CycN-K6** and **CycN-K7**

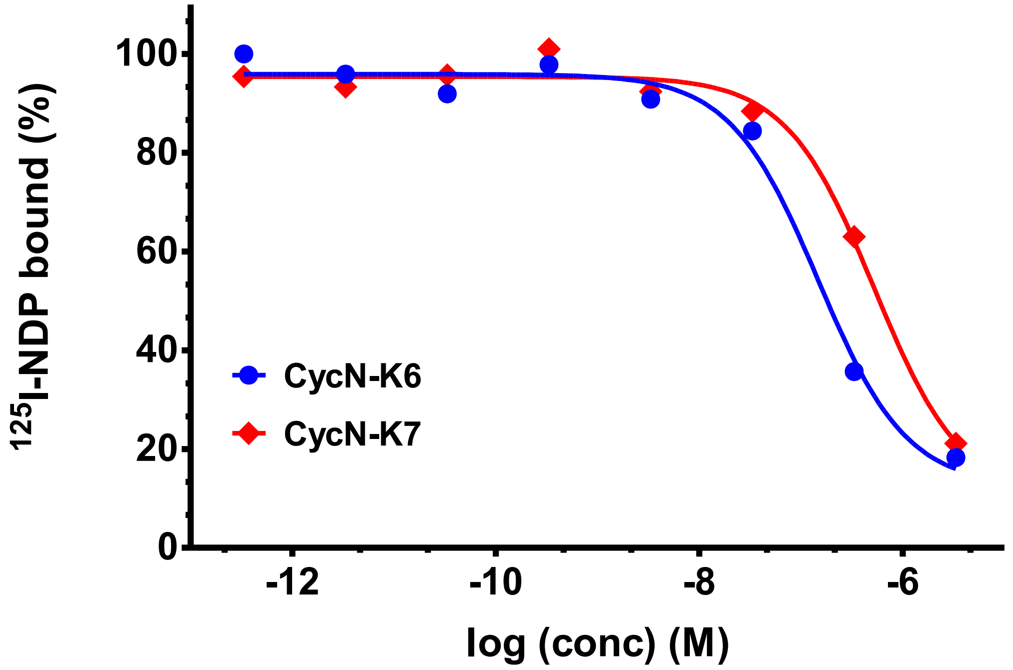

Figure S1. Competitive binding curves for **CycN-K6** (blue line) and **CycN-K7** (red line)

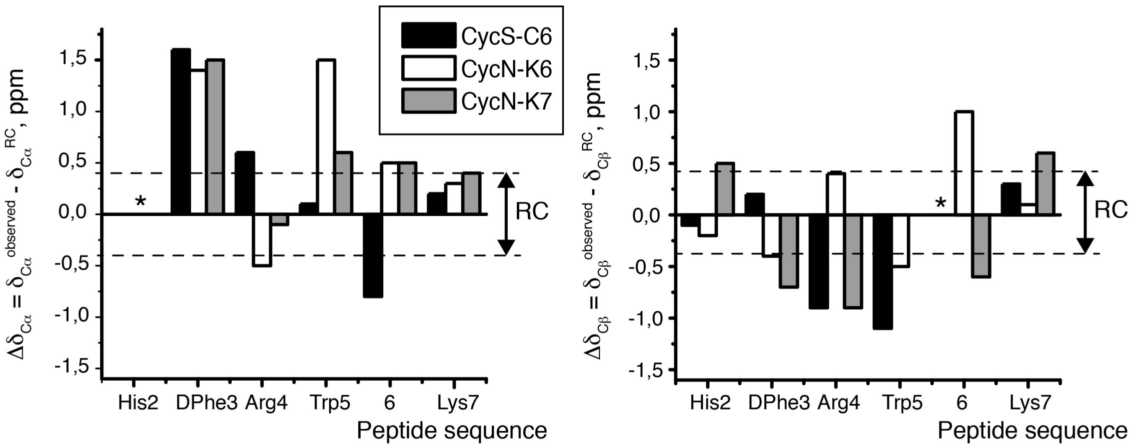

Figure S2. Bar plots showing the $\Delta\delta_{C\alpha}$ ($\Delta\delta_{C\alpha} = \delta_{C\alpha}^{observed} - \delta_{C\alpha}^{RC}$, ppm), and $\Delta\delta_{C\beta}$ ($\Delta\delta_{C\beta} = \delta_{C\beta}^{observed} - \delta_{C\beta}^{RC}$, ppm) values as a function of sequence for peptides **CycS-C6** (black bars; Morais et al., 2012), **CycN-K6** (white bars), and **CycN-K7** (grey bars) in aqueous solution at pH 2.5 and 5 ºC. Residue 6 is Cys in **CycS-C6** and Lys in **CycN-K6** and **CycN-K7**. Dashed lines indicate the random coil (RC) ranges. An asterisk (*) indicates that the C_α of His2 residues could not be measured (see Tables S1 and S2), and that the available random coil values are not appropriate for the C_β of Cys6 in **CycS-C6**, because the pNO_2-benzoic acid affects its chemical shift (see Figure 1). Random coil values were taken from Wishart et al., 1995.

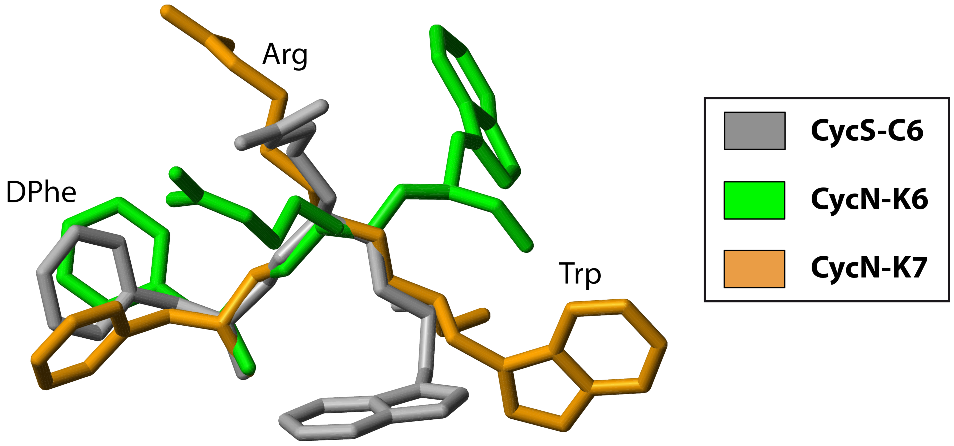

Figure S3. Overlay of representative structures for peptides **CycS-C6** (grey) **CycN-K6** (green) and **CycN-K7** (orange) highlighting the relative dispositions of DPhe, Arg and Trp side chains.

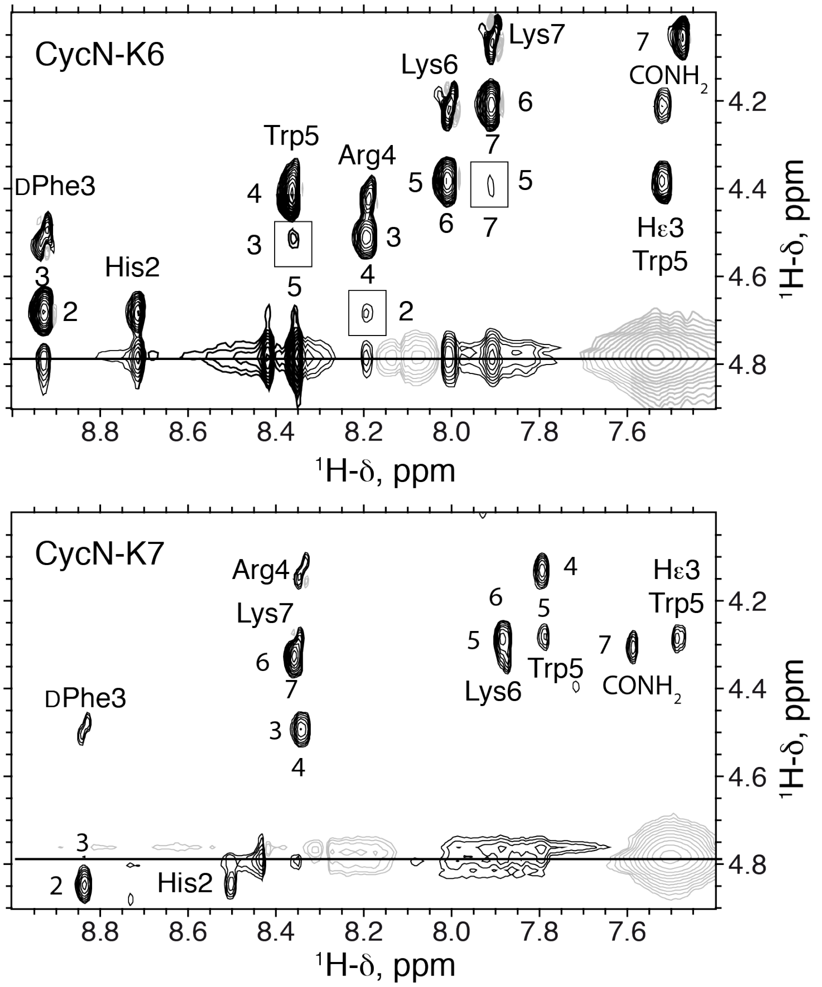

Figure S4. ^{1}H,^{1}H-ROESY spectral region of peptides **CycN-K6** and **CycN-K7** in aqueous solution (H_2O/D_2O 9:1 v/v) at pH 2.5 and 25 ºC. Intraresidual H_α-NH cross-peaks are labelled with the residue name and number, and the sequential H_α-NH(i,i+1) and the non-sequential H_α-NH(i,i+2) with the residue number for the Hα at the left or the right, and for the HN above or below. $CONH_2$ indicates the C-terminal amide. The intraresidual H_α-$H_{\varepsilon3}$ Trp5 cross-peak is also seen in this region. Non-sequential cross-peaks are boxed. The horizontal line at about 4.78 ppm comes from the H_2O solvent.

Table S1. ^{1}H, ^{13}C and ^{15}N chemical shifts (ppm, from DSS) for peptide **CycN-K6** in H_2O/D_2O 9:1 v/v at pH 2.5 and 25 ºC. "nd" stands for not determined.

Residue	^{15}N	HN	$^{13}C_{\alpha}$	$C_{\alpha}H$	$^{13}C_{\beta}$	$C_{\beta}H$	Others
pNO_2-benzoic acid 1	----	-----	-----	-----	-----	-----	$C_{\delta1}H$ 8.42;<br>$C_{\delta2}H$ 6.85;<br>$C_{\varepsilon2}H$ 8.12
H2	119.5	8.72	nd	4.68	28.8	3.23, 3.23	$C_{\delta2}H$ 7.15;<br>$C_{\varepsilon1}H$ 8.36
DF3	125.6	8.93	59.1	4.51	39.2	2.75, 3.08	$C_{\delta\delta'}H$ 7.19, 7.19;<br>$C_{\varepsilon\varepsilon'}H$ 7.36, 7.36;<br>$C_{\zeta}H$ nd
R4	121.2	8.20	55.5	4.42	31.3	1.48, 1.69	$^{13}C_{\gamma}$ 27.0;<br>$C_{\gamma}H$ 1.02, 1.22;<br>$^{13}C_{\delta}$ 43.7;<br>$C_{\delta}H$ 2.48, 2.82;<br>$^{15}N_{\varepsilon}$ 84.6; $N_{\varepsilon}H$ 6.90<br>$N_{\eta}H$ 6.52
W5	123.0	8.37	59.0	4.38	29.1	3.03, 3.26	$C_{\delta1}H$ 7.04;<br>$^{15}N_{\varepsilon1}$ 129.4;<br>$N_{\varepsilon1}H$ 10.00;<br>$C_{\varepsilon3}H$ 7.52;<br>$C_{\zeta3}H$ 7.11;<br>$C_{\eta2}H$ 7.17;<br>$C_{\zeta2}H$ 7.36
K6	122.2	8.01	56.7	4.21	34.1	1.66, 1.80	$^{13}C_{\gamma}$ 26.4;<br>$C_{\gamma}H$ 1.37, 1.45;<br>$^{13}C_{\delta}$ 30.6;<br>$C_{\delta\delta'}H$ 1.62, 1.67;<br>$^{13}C_{\varepsilon}$ 45.9;<br>$C_{\varepsilon\varepsilon'}H$ 3.30, 3.36;<br>$^{15}N_{\zeta}$ nd; $N_{\zeta}H$ nd
K7	121.3	7.91	56.5	4.06	33.2	1.68, 1.77	$^{13}C_{\gamma}$ 25.0;<br>$C_{\gamma}H$ 1.40, 1.40;<br>$^{13}C_{\delta}$ 29.4;<br>$C_{\delta\delta'}H$ 1.66, 1.66;<br>$^{13}C_{\varepsilon}$ 42.4;<br>$C_{\varepsilon\varepsilon'}H$ 2.99, 2.99;<br>$^{15}N_{\zeta}$ 68.8;<br>$N_{\zeta}H_3$ 7.54
$CONH_2$	108.2	7.07, 7.48					

Table S2. ^{1}H, ^{13}C and ^{15}N chemical shifts (ppm, from DSS) for peptide **CycN-K7** in H_2O/D_2O 9:1 v/v at pH 2.5 and 25 ºC. "nd" stands for not determined.

Residue	^{15}N	HN	$^{13}C_\alpha$	$C_\alpha H$	$^{13}C_\beta$	$C_\beta H$	Others
pNO_2 benzoic acid 1	----	-----	-----	-----	-----	-----	$C_{\delta1}H$ 8.30;<br>$C_{\delta2}H$ 6.69;<br>$C_{\varepsilon2}H$ 7.86
H2	117.1	8.51	nd	4.85	29.5	3.24, 3.31	$C_{\delta2}H$ 7.15;<br>$C_{\varepsilon1}H$ 8.43
DF3	124.4	8.84	59.2	4.49	38.9	2.83, 3.00	$C_{\delta\delta'}H$ 7.20, 7.20;<br>$C_{\varepsilon\varepsilon'}H$ 7.34, 7.34;<br>$C_\zeta H$ 7.32
R4	124.6	8.34	55.9	4.13	30.0	1.41, 1.60	$^{13}C_\gamma$ 26.9;<br>$C_\gamma H$ 0.94, 1.08;<br>$^{13}C_\delta$ 43.3;<br>$C_\delta H$ 2.71, 2.85;<br>$^{15}N_\varepsilon$ 84.3;<br>$N_\varepsilon H$ 6.94<br>$N_\eta H$ 6.55
W5	122.5	7.80)	58.1	4.29	29.6	2.99, 3.04	$C_{\delta1}H$ 6.80;<br>$^{15}N_{\varepsilon1}$ 129.4;<br>$N_{\varepsilon1}H$ 10.00;<br>$C_{\varepsilon3}H$ 7.48;<br>$C_{\zeta3}H$ 7.07;<br>$C_{\eta2}H$ 7.14;<br>$C_{\zeta2}H$ 7.34
K6	123.3	7.89	56.7	4.33	32.5	1.57, 1.68	$^{13}C_\gamma$ 24.6;<br>$C_\gamma H$ 1.25, 1.30;<br>$^{13}C_\delta$ 29.1;<br>$C_{\delta\delta'}H$ 1.61, 1.61;<br>$^{13}C_\varepsilon$ 42.2;<br>$C_{\varepsilon\varepsilon'}H$ 2.94, 2.94;<br>$^{15}N_\zeta$ 68.0;<br>$N_\zeta H_3$ 7.48
K7	124.4	8.36	56.6	4.31	33.7	1.85, 1.90	$^{13}C_\gamma$ 25.4;<br>$C_\gamma H$ 1.48, 1.56;<br>$^{13}C_\delta$ 30.0;<br>$C_\delta H$ 1.65, 1.65;<br>$^{13}C_\varepsilon$ 45.3;<br>$C_\varepsilon H$ 3.27, 3.39;<br>$^{15}N_\zeta$ nd; $N_\zeta H$ nd
$CONH_2$	107.1	7.11, 7.59					

Table S3. NOEs between pNO_2-benzoic acid unit and its adjacent residues His and Lys in peptides **CycN-K6** and **CycN-K7**.

**Proton from**		**Peptide**	
**pNO_2-benzoic acid**	**Residue**	**CycN-K6**	**CycN-K7**
δ1	HN His 2	Strong	Strong
δ1	ββ' His 2	Medium	Not detected
δ2	εε' Lys 6	Strong	-----
δ2	δδ' Lys 6	Strong	-----
δ2	εε' Lys 7	-----	Strong
δ2	δδ' Lys 7	-----	Strong

Table S4. $^3J_{\alpha N}$ coupling constants (Hz) measured in 1D ^1H-NMR spectra of peptides **CycS-C6** (Morais et al., 2012), **CycK6** and **CycK7** in aqueous solution (H_2O/D_2O v/v) at pH 2.5 at 25 ºC. Experimental error: ± 0.4 Hz. [a] Accurate value could not be measured because of signal overlap.

	**$^3J_{\alpha N}$ coupling constant (Hz) for residue**					
**Peptide**	His 2	DPhe 3	Arg 4	Trp 5	Cys/Lys 6	Lys 7
**CycS-C6**	7.9	4.8	7.0	7.1	7.0	7.1
**CycN-K6**	4.2	6.6	8.7	~6.5[a]	7.6	6.5
**CycN-K7**	6.0	6.2	~8.6[a]	6.1	~8.1[a]	7.7

Table S5. Structural statistics of the 20 best NMR structures of peptides **CycN-K6** and **CycN-K7** in aqueous solution (H_2O/D_2O 9:1 v/v) at pH 2.5 at 25 ºC.

	**CycN-K6**	**CycN-K7**
**NOE distance constraints**		
Short-range distances (i-j) ≥ 1	80	71
Medium-range distances (i-j) < 5	3	3
Long-range distances (i-j) ≥ 5	0	0
Total	83	74
Stereospecific assignment	1	2
**Final CYANA target function value** ($Å^2$)	4.5 E-5	1.4 E-7
**RMSD to lowest target function structure (Å)** [a]		
Backbone atoms + heavy atoms of linker belonging to the cycle	0.7	0.8
All heavy atoms	2.8	1.8
**Ramachandran plot statistics**		
Most favorable regions (%)	70.0	60.0
Additional allowed regions (%)	30.0	40.0
Generously allowed regions (%)	0.0	0.0
Disallowed regions (%)	0.0	0.0

[a] The corresponding RMSD values for **CycS-C6** structure (Morais et al., 2012) are 0.4 Å for backbone + linker, and 1.9 Å for all heavy atoms.

References

Morais, M.; Raposinho, P.D.; Oliveira, M.C.; Pantoja-Uceda, D.; Jimenez, M.A.; Santos, I.; Correia, J.D.G. NMR structural analysis of MC1R-targeted rhenium(i) metallopeptides and biological evaluation of Tc-99m(i) congeners. Organometallics 2012, 31, 5929-5939.

Wishart, D.S.; Bigam, C.G.; Holm, A.; Hodges, R.S.; Sykes, B.D. H-1, C-13 and N-15 random coil NMR chemical-shifts of the common amino-acids. 1. Investigations of nearest-neighbor effects. J Biomol NMR 1995, 5, 67-81.
